# The Feasibility of Using Acoustic Markers of Speech for Optimizing Patient Outcomes during Randomized Amplitude Variation in Deep Brain Stimulation: A Proof of Principle Methods Study

**DOI:** 10.3389/fbioe.2015.00098

**Published:** 2015-07-14

**Authors:** Adam P. Vogel, Hugh J. McDermott, Thushara Perera, Mary Jones, Richard Peppard, Colette M. McKay

**Affiliations:** ^1^The Bionics Institute of Australia, Melbourne, VIC, Australia; ^2^Speech Neuroscience Unit, The University of Melbourne, Melbourne, VIC, Australia; ^3^Department of Neurodegeneration, Hertie Institute for Clinical Brain Research, University of Tübingen, Tübingen, Germany; ^4^Department of Audiology and Speech Pathology, The University of Melbourne, Melbourne, VIC, Australia; ^5^Department of Medical Bionics, The University of Melbourne, Melbourne, VIC, Australia; ^6^Department of Neurology, St Vincent’s Hospital, Melbourne, VIC, Australia

**Keywords:** side effects, optimization, dysarthria, acoustic analysis, speech, brain stimulation

## Abstract

**Background:**

Deep brain stimulation (DBS) is an effective treatment for reducing symptoms of tremor. A common and typically subjectively determined adverse effect of DBS is dysarthria. Current assessment protocols are driven by the qualitative judgments of treating clinicians and lack the sensitivity and objectivity required to optimize patient outcomes where multiple stimulation parameters are trialed.

**Objective:**

To examine the effect of DBS on speech in patients receiving stimulation to the posterior sub-thalamic area (PSA) via randomized manipulation of amplitude parameters.

**Methods:**

Six patients diagnosed with tremor receiving treatment via DBS of the PSA were assessed in a double-blinded, within-subjects experimental protocol. Amplitude (i.e., voltage or current) was randomly adjusted across 10 settings, while speech samples (e.g., sustained vowel, counting to 10) were recorded to identify the patient-specific settings required for optimal therapeutic benefit (reduced tremor) with minimal adverse effects (altered speech). Speech production between stimulation parameters was quantified using acoustic analysis.

**Results:**

Speech changed as a response to DBS but those changes were not uniform across patients nor were they generally in line with changes in amplitude with the exception of reduced vocal control and increased mean silence length in two patients. Speech outcomes did not correlate with changes in tremor.

**Conclusion:**

Intra-individual changes in speech were detected as a response to modified amplitude; however, no clear pattern was observed across patients as a group. The use of objective acoustic measures allows for quantification of speech changes during DBS optimization protocols, even when those changes are subtle and potentially difficult to detect perceptually.

## Introduction

Deep brain stimulation (DBS) is well established as a safe and effective treatment option for reducing tremor severity where drug therapy is ineffective (Plaha et al., 2004; Blomstedt et al., 2010). Aside from perioperative complications (e.g., intra-cranial hemorrhage) (Hariz, 2002) and hardware-related problems (e.g., electrode misplacements, infections, and device malfunction) (Carvallo et al., 2011), two issues relating to stimulation and optimization inhibit the utility of the technique. First, the possible combinations of stimulus parameter settings are too great for clinical-based exploration in individuals, often leading to the use of default parameters. Second, a common and typically unquantified (i.e., assessed subjectively) adverse effect of DBS is dysarthria (neuromuscular speech disorder) (Skodda et al., 2011; Tripoliti et al., 2011).

The efficacy of DBS is partly dependent on the capacity of clinicians to determine optimal stimulation settings. Yet, qualitative assessment protocols do not allow for precise comparison between the effects of stimulus parameter settings. They also limit the capacity of clinicians to evaluate the comparative influence of tremor and speech effects when determining a clinical endpoint. Historically, empirical investigations of speech production in DBS have utilized perceptual assessment practices, where a clinician listens to the speech of a patient in order to judge its quality (Murata et al., 2003; Plaha et al., 2004; Farrell et al., 2005). However, the limitations of subjective evaluation of speech (i.e., bias and error) are well known (Kent, 1996), including limited capacity of listeners to determine the size of change from one production to the next, the influence of experience on the capacity of a listener to identify (and quantify) changes in speech, and the limited inter- and intra-rater reliability of auditory-perceptual judgment (Vogel and Maruff, 2014). An objective alternative to listener-based observation is acoustic analysis of speech, which provides objective measurement of speech output through the study of the physical properties of the speech signal. By quantifying changes in speech that may be associated with DBS, the clinician/researcher is able to determine the size and nature of any side-effects alongside the desired primary outcome of tremor reduction.

Speech does not occur in all patients receiving DBS. In patients with essential tremor receiving DBS, reports of stimulation-induced dysarthria occur in 10% of cases (Flora et al., 2010). Perhaps more importantly, changes in speech that can occur following DBS are highly variable (Xie et al., 2012). Speech outcomes appear to differ depending on site of stimulation, etiology of disorder, site of lesion, severity of tremor, and methods used to measure speech production (Deuschl et al., 2006; Tripoliti et al., 2008). Documented changes to speech after DBS include, but are not limited to, increases in voice onset time and decreased rate of speech (Barbe et al., 2014), inappropriate voicing (Karlsson et al., 2012), decreased maximum phonation time (Pinto et al., 2014), longer maximum phonation time and faster rate of speech (Gentil et al., 2003), reduced intelligibility (Törnqvist et al., 2005; Tripoliti et al., 2008; Åström et al., 2010; Pinto et al., 2014), and improved vocal quality and control (D’Alatri et al., 2008).

Few studies have combined objective measures of speech and systematic modification of DBS stimulus settings. Where objective (acoustic) measures were used, the majority of studies have evaluated the effect of DBS via an ON/OFF stimulation protocol (Gentil et al., 2003; Van Lancker Sidtis et al., 2010; Karlsson et al., 2012). Where researchers have altered DBS electrical parameters beyond a simple on/off paradigm, most have utilized perceptual (subjective) assessments of speech (Törnqvist et al., 2005; Tripoliti et al., 2008; Åström et al., 2010). A recent study, which investigated the therapeutic benefit of differentially stimulating two contacts on the same electrode with the aim of reducing stimulation-induced dysarthria, used a combination of acoustic, patient self-report, and listener ratings to measure changes in speech (Barbe et al., 2014). These authors appear to be the first to have published data on systematically modifying DBS settings for the purpose of alleviating dysarthria. Through the use of voice onset time (objective) and patient-completed visual analog scales (subjective), their study showed that dysarthric side effects can be reduced using individualized current shaping on two active DBS electrodes. Their proof of principle study demonstrated the potential value of adjusting stimulation parameters for individual patients, although it was limited by the use of only one objective measure of speech.

In the experiments reported below, we objectively tracked changes in speech resulting from randomly altered stimulus amplitude in patients with tremor (either essential, cerebellar intention, or dystonic tremor types) receiving stimulation to the posterior sub-thalamic area (PSA). Patients and experimenters assessing speech were blinded to stimulus settings. The protocol aimed to capture changes in speech resulting from altered stimulus amplitude using a protocol proven to be stable and sensitive for monitoring change over time. We aimed to identify patient-specific settings that resulted in optimal therapeutic benefit (reduced tremor) with minimal adverse effects (altered speech). Given the heterogeneity of speech outcomes from earlier work, it was hypothesized that the optimal stimulus level for tremor suppression would not correspond to optimal speech outcomes.

## Patients and Methods

Six adults diagnosed with essential tremor, cerebellar intention tremor, or dystonic tremor and receiving treatment via DBS of the PSA were recruited from a neurology clinic in Melbourne, VIC, Australia. Demographic and implant information is provided in Table 1. Patients were included in the study if they presented with a confirmed diagnosis of tremor, had undergone DBS surgery with bilateral stimulation of the PSA, were aged between 18 and 80 years, and had English as their first language. Patients were excluded if they reported speech impairment prior to the onset of tremor or DBS treatment. Approval to conduct this study was obtained from the Royal Melbourne Hospital Ethics Committee. Written informed consent for research was obtained from all patients participating in the study.

**Table 1:** **Participant demographic and implant information**.

Patient	Diagnosis	Sex	Age (years)	Months post DBS implantation	Years since onset of symptoms	Model/device	Unilateral/bilateral	Clinically set pulse duration (**μ**s)	Clinically set frequency (Hz)	Clinically set amplitude	Randomized amplitude settings by patient^c^
1	ET	M	72	4	18	St Judes Brio ActiveTip 3 mm leads^a^	Bilateral	90	130	2.5 (bilateral)	0.0, 0.75, 1.5, 2.2, 3.0, 3.5, 4.0
2	ET	F	48	17	20	Medtronic activa RC model 3387^b^	Bilateral	90	130	2.5 (bilateral)	0.0, 1.0, 1.5, 2.0, 2.5, 3.0, 3.5
3	Cerebellar intention tremor	F	71	15	35	St Jude Libra ActiveTip 3 mm leads^a^	Bilateral	90	130	1.4 (L), 0.6 (R)	0.0, 0.6, 1.5, 2.1, 2.8, 3.5, 4.2
4	Dystonic tremor	F	47	6	25	Medtronic activa RC model 3387^b^	Bilateral	90	130	3.5 (L), 2.3 (R)	0.0, 1.5, 1.8, 2.1, 2.4, 2.7, 3.0
5	ET	M	56	16	18	Medtronic activa RC model 3387^b^	Bilateral	90	130	2.4 (L), 3.3 (R)	3.3, 3.8, 4.4, 5.0, 5.4, 5.8^d^
6	ET	F	44	15	30	Medtronic activa RC model 3387^b^	Bilateral	60	150	2.3 (bilateral)	0.0, 1.5, 1.9, 2.4, 3.0, 3.6, 4.3

*^a^Device measured in milliamps*.

*^b^Device measured in volts*.

*^c^Pulse duration and frequency set to 90 and 130, respectively, for all patients and conditions*.

*^d^Participant became emotionally labile when lower voltages were attempted*.

*ET, essential tremor; L, left; R, right; Hz, Hertz; mA, milliamps; M, male; F, female*.

*Patient 6 treated with clonazepam 0.5 mg/day in addition to DBS. All patients implanted bilaterally*.

### Surgery

Preoperative MRI and stereotactic CT images were fused using the StealthStation Surgical Navigation System (Medtronic, Minneapolis, MN, USA) to plan lead trajectory. Target coordinates for the PSA were determined as a point 2–3 mm lateral to the equator of the red nucleus, halfway to the sub-thalamic nucleus, 4–6 mm below the intercommissural plane. The trajectory was planned to avoid vessels, sulci, and ventricles. We performed intraoperative microrecording and microstimulation (Leadpoint-system, Medtronic) to verify the target coordinates, and the most-ventral contact of the DBS lead was positioned at this point. Table 1 lists the neurostimulator and lead types for each patient. All patients were implanted bilaterally. Following surgery, electrode placement was verified by an independent neurosurgeon using postoperative CT fused with the preoperative MRI.

### Procedure

A double-blinded, within-subjects experimental design was employed. Patients and assessors were blinded to the stimulation condition. Electrical stimulation parameters (i.e., voltage or current levels) were systematically adjusted following a random order of testing over the course of one session. Stimulation parameters were switched after speech and movement tasks were completed.

### Stimulus amplitude manipulation

The clinically set values of the stimulation parameters (determined on a previous visit by the consultant neurologist and specialist nurse) were used as reference values around which stimulation variations were made (see Table 1). The Medtronic devices used by a subset of these subjects control the level of stimulus amplitude using voltage, whereas the St Jude devices use current. Here, we have described both voltage and current as amplitude. The stimulator controlling the most-affected side was adjusted first, while the second side was turned off. Keeping reference values of frequency (130 Hz) and pulse duration (90 μs) fixed, amplitude was manipulated so that the levels at which tremor suppression began (threshold) and side effects (e.g., general tingling sensations, visual disturbances beyond typical function, emotional lability) began to appear, were established using patient feedback and clinical observation. A series of amplitude steps between threshold and side-effect onset were then selected for use in the assessments, in addition to the device-off condition. For example, where the tremor suppression threshold was 0.75 V, and side-effect onset was 4 V, the series of voltage steps was (0.0, 0.75, 1.5, 2.2, 3.0, 3.5, and 4.0 V). The amplitude steps were presented in random order. No data were collected in the first minute following each parameter adjustment to facilitate adaptation to the new value. After this period, both tremor and speech assessments were administered. Following the measures with unilateral stimulation, the first-side amplitude was fixed at the clinically observed optimal amplitude for tremor suppression, and the second device turned on. The threshold for tremor suppression and side effect were then established for the second side (while leaving the first-side on) and three amplitudes were selected that spanned the range between threshold and side effect. The same tremor and speech measures were obtained for these three bilateral conditions.

### Patient evaluation

#### Tremor

During the tremor examination, patients were required to perform two manual tasks in order to assess postural and kinetic tremors. Tasks included sustained bilateral arm extension, and a bilateral finger–nose–finger maneuver with each arm (Elble et al., 2012) (while verbally counting to 10). Tremor severity was assessed during these tasks on a scale of 0–10 through consensus rating by the consultant neurologist and a specialist nurse. Each limb was assessed separately, and the two scores averaged for data analysis.

#### Speech Analyses

Speech samples were acquired using a standard laptop PC coupled with an external sound card (model UA-25, Roland Corporation, Shizuoka, Japan) and an AKG 520C condenser microphone (AKG Acoustics GmbH, Vienna, Austria). Patients performed three speech production tasks in each condition: (i) producing a sustained vowel /a:/ for 6 s; (ii) counting from 1 to 10; and (iii) reading a phonetically balanced paragraph, the “Grandfather passage” (Van Riper, 1963). Patients were asked to practice the tasks once before recording commenced to reduce unfamiliarity effects often observed in repeated trials with brief inter-recording intervals (Vogel et al., 2011; Vogel and Maruff, 2014).

Quantitative data were extracted from the sound recordings using automated scripts written for use with freely available software, Praat (Boersma, 2001), to generate measures of timing, vocal control, and voice quality. Measures of timing were obtained from recordings of speech produced in the counting and reading tasks, and included speech rate (SRATE, syllables/second), mean silence time (SMEAN, seconds), variability of silence length (VSIL, seconds), and percentage of silence in a sample (PSIL). Timing measures were automatically calculated using methods designed to identify “silences” within a speech sample based on the intensity contour using a modified version of techniques described by Rosen et al. (2010) and implemented by Vogel et al. (2012, 2014). Three thresholds were defined to identify silences from the intensity contour: (a) *Intensity threshold*, (b) *minimum silence duration* (15 ms), and (c) *minimum speech duration* (30 ms). Silence segments were defined as the parts of the intensity contour that fell below the *intensity threshold*. Silence sections that were shorter than 15 ms were classed as speech and concatenated with the adjacent speech sections. Speech sections that were shorter than 30 ms were classed as silences and concatenated with the adjacent silences. The *intensity threshold* was set to 0.65 of the distance between the reference intensity (equal to 0.95 of the maximum intensity) and floor intensity (minimum). Reference intensity selection of 0.95 of the maximum intensity has been found more robust than use of the maximum, median, or modal intensities due to irregular bursts of energy that often occur with sporadically loud syllables or short phrases in spontaneous speech (e.g., emphatic stress). Visual inspection of the spectrum has shown that 0.95 of the maximum intensity represents the typical intensity of syllable peaks, whereas maximum intensity reflects a single observation interval and is less reliable than use of the reference intensity threshold described. The timing measures derived from this method included total silence time, total speech time, the percentage of silence in the sample, and speech rate (number of syllables/total signal time).

Fundamental frequency (*f*_0_) and coefficient of variability (CoVs) were derived from the sustained vowel task using an automated PRAAT script (Vogel et al., 2009). *f*_0_ calculations are made in PRAAT by employing a user-supplied estimate of the window length for acoustic analysis. To determine window length, two primary program parameters are taken into consideration: *time step* and *pitch floor*. *Time step* is a measurement interval (frame duration) parameter measured in seconds, and is calculated by dividing 0.75 by the *Pitch floor*. For example, if the *Pitch floor* is set to 75 Hz, then the *Time step* equals 0.01 s (0.75/75), specifying 100 pitch values to be computed by PRAAT per second. *Pitch floor* is used to specify the length of the acoustic analysis window, and also represents the lowest fundamental frequency (*f*_0_) that can be measured within each speech sample. To accurately calculate pitch, the analysis window must be long enough to detect three periods of the pitch frequency to be identified. For example, in order to identify a *Pitch floor* of 75 Hz, the effective analysis window will be 3/75, or 0.04 s long. Increasing the *Time step* will speed up the editor window; however, it can lead to under sampling of the pitch and formant curves, which could influence the accuracy of the selected acoustic measures. *Pitch ceiling* is a program parameter used at the post-processing stage to ignore candidates above the prescribed setting based on prior research and experience. This process promotes the most efficient use of available data and is based on empirical findings detailed in Vogel et al. (2009), which demonstrated the reliability and validity of the described methods. Data provided in this report calculated *f*_0_ using generic window lengths to expedite batch processing of the speech samples for both male and female populations. Pitch range settings for males encompassed low *Pitch floors* (70 Hz) and a mid-level *Pitch ceiling* (250 Hz). *Pitch floor* settings of 100 Hz and *ceiling* setting of 300 Hz were used for female speakers.

Voice quality was determined using harmonics-to-noise ratio (HNR) derived from Praat. The sustained vowel task was chosen to evaluate voice quality and control as it provides better classification of disordered voicing compared to connected speech samples (Parsa and Jamieson, 2001). The focus on measures of timing and laryngeal control was designed to provide a proxy of overall dysarthria severity using easily acquired objective measures of speech. These measures have demonstrated the sensitivity to change and impairment in both healthy and pathological groups (e.g., fatigue, noise, hereditary ataxia, Huntington’s disease) (Vogel and Maruff, 2008; Vogel et al., 2010, 2011, 2012, 2014; Mundt et al., 2012; Yiu et al., 2015). Importantly, the stability of the chosen measures has been established in a series of within-subjects experiments over short-, medium-, and long-term inter-recording intervals (Vogel et al., 2011; Vogel and Maruff, 2014).

Overall improvements in speech were determined via higher values of HNR (improved voice quality), lower values of *f*_0_ CoV (improved vocal control), and the following measures of timing efficiency and control: lower mean silence length, increased speech rate, reduced silence length variability, and decreased percent silence. Reduced mean silence length can indicate more natural sounding speech, because, as a speaker transitions from one phonemic element to the next, they are able to continue voicing with shorter breaks. Decreased mean silence length combined with reduced silence length variability and decreased percent silence result in increased speech rate and overall improved efficiency (Yiu et al., 2015).

## Results

**Figure 1:**
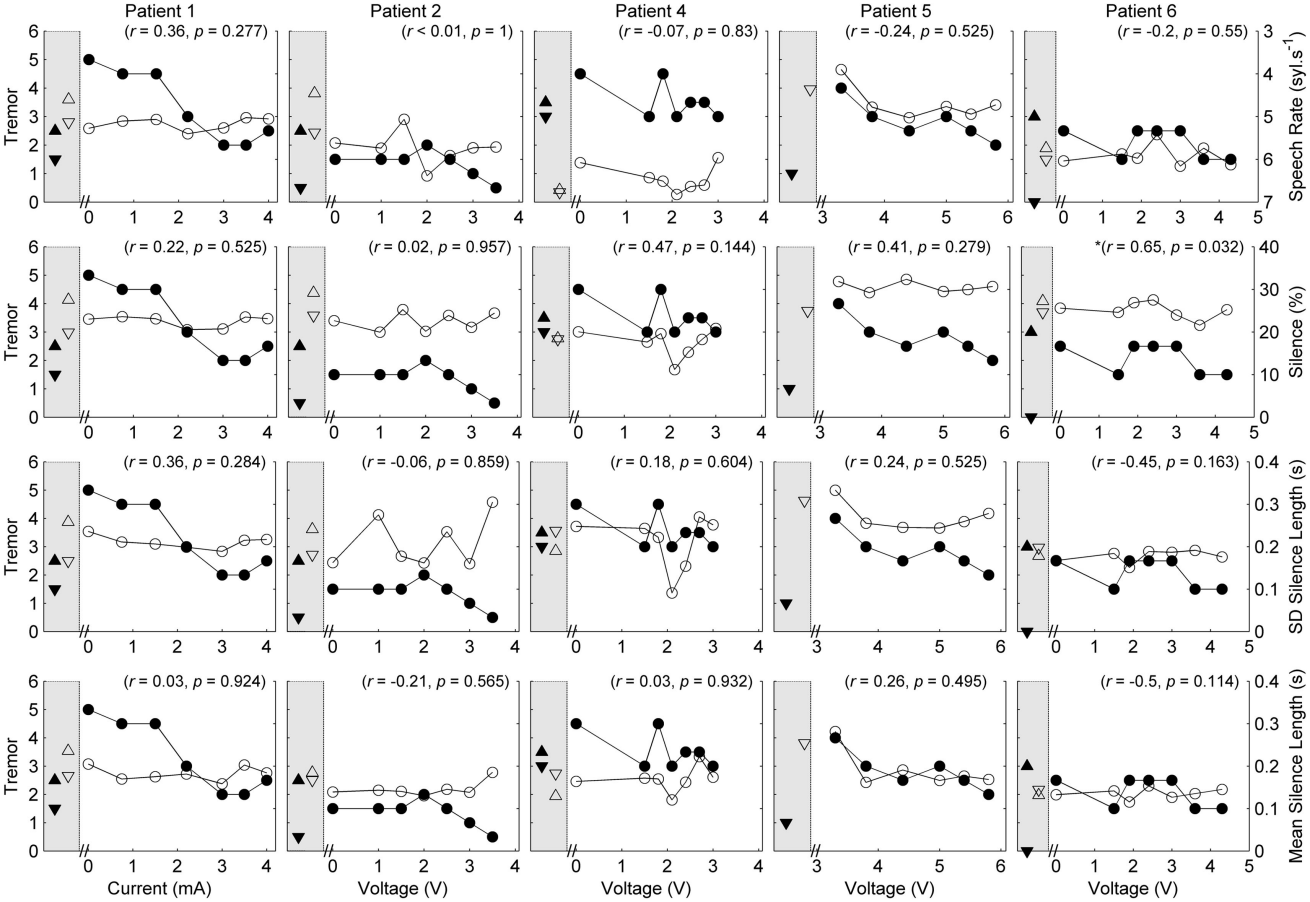
**Speech and tremor data during the reading passage**. Closed circles, tremor severity; open circles, speech timing measures; in the left shaded area of each panel are the clinical baseline tremor ratings (up closed triangle), the best tremor rating obtained in the study (down closed triangle). Both baseline and best performance ratings were obtained with bilateral stimulation, along with the corresponding speech scores for the same conditions (up open triangle for clinical baseline and down closed triangle for best performance). For all measures, values closer to the *x*-axis were considered an improvement. *r* values reflect correlation between speech and tremor.

**Figure 2:**
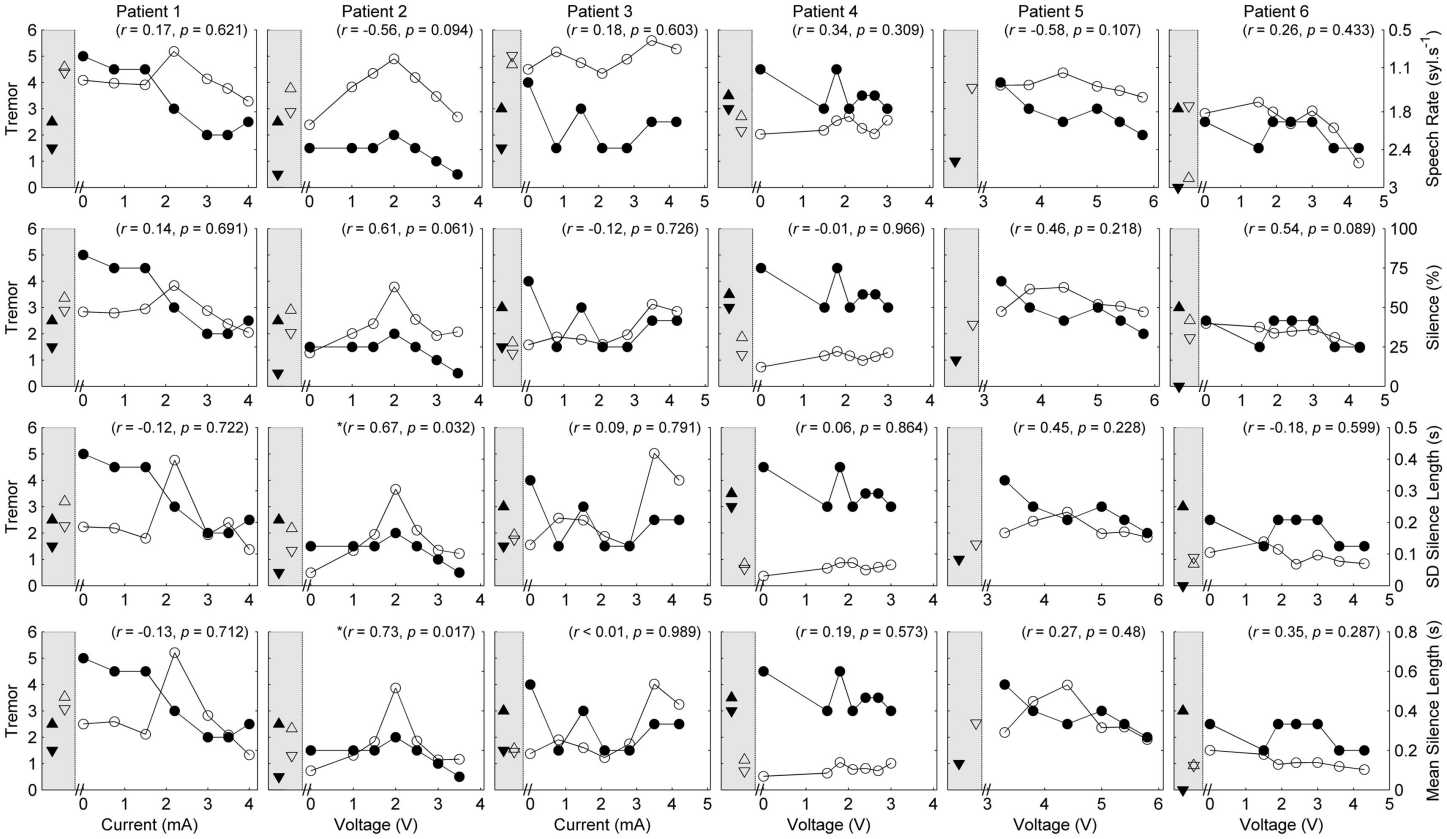
**Speech and tremor data during the counting task**. Closed circles, tremor severity; open circles, speech timing measures; in the left shaded area of each panel are the clinical baseline tremor ratings (up closed triangle), the best tremor rating obtained in the study (down closed triangle). Both baseline and best performance ratings were obtained with bilateral stimulation, along with the corresponding speech scores for the same conditions (up open triangle for clinical baseline and down closed triangle for best performance). For all measures, values closer to the *x*-axis were considered an improvement. *r* values reflect correlation between speech and tremor.

**Figure 3:**
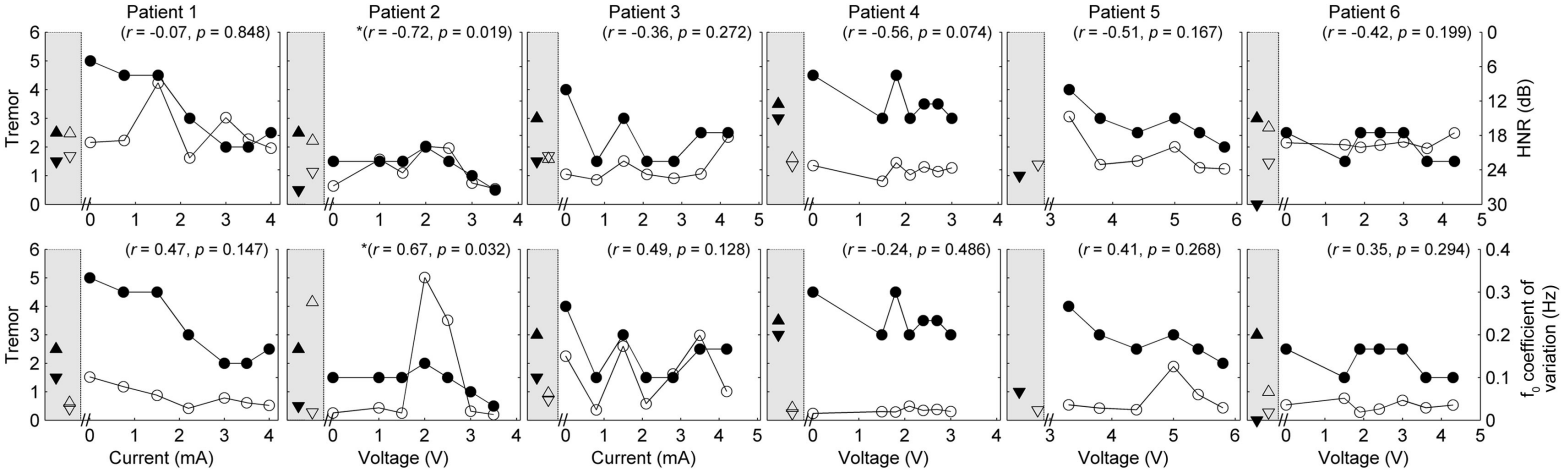
**Speech and tremor data during the sustained vowel task**. Closed circles, tremor severity; open circles, voice quality and control measures; in the left shaded area of each panel are the clinical baseline tremor ratings (up closed triangle), the best tremor rating obtained in the study (down closed triangle). Both baseline and best performance ratings were obtained with bilateral stimulation, along with the corresponding speech scores for the same conditions (up open triangle for clinical baseline and down closed triangle for best performance). Note that the HNR scale is reversed to show improvements in performance reflected in decreased values on the figure. *r* values reflect correlation between speech and tremor.

Five of the six patients completed speech/movement tasks at all stimulus amplitudes. Patient 5 did not tolerate reduction of stimulation, completing 9/10 experimental conditions. P5 did not have a clinical baseline measured, as she commenced the protocol with one of her devices turned off. Figures 1 and 2 show the effects of unilateral current or voltage manipulation on tremor severity and speech timing measures for the reading and counting tasks, respectively. Figure 3 shows the voice quality and control measures along with the same tremor measures as Figures 1 and 2. Data on baseline performance are also displayed to provide comparative information on the effects of stimulation. Data indicate the effect of stimulus amplitude on tremor severity and speech quality differed among subjects (Figures 1–3).

### Response to DBS: Tremor

Where data were broken down into three classes (Off stimulation; Maximum amplitude; Amplitude threshold – where tremor suppression begun), one-tailed *t*-tests showed that tremor severity was significantly reduced for stimulus amplitudes at the side-effect threshold (*t* = 5.477, *p* = 0.003) and the tremor suppression threshold (*t* = 2.557, *p* = 0.03), compared to the device-off condition. There was no significant difference between tremor at the tremor suppression threshold and the side-effect threshold (*t* = 1.348, *p* = 0.12). Two-tailed paired *t*-tests comparing speech measures at the same three unilateral stimulus amplitudes did not show any significant group effects of stimulus amplitude (*p* > 0.05).

In each case [except Patient 5 (P5) where data were not available], tremor severity decreased with introduction of DBS (comparing the device-off condition, with the optimum bilateral condition of the study, as illustrated by closed down-triangles in Figures 1–3). For all subjects except P5, the optimal experimental tremor reduction was equal to or better than the clinical baseline tremor reduction (comparing the down and up closed triangles). In two cases (P2 and P6), the clinical baseline measure showed worse or similar tremor to the “off” condition.

### Correlation between amplitude, tremor, speech, and voice measures

Pooling data from all patients revealed no significant correlations between tremor and speech/voice measures. Covariance of acoustic measures from all patients revealed strong and significant relationships between SMEAN and VSIL (ρ = 0.915, *p* < 0.001), PSIL (ρ = 0.828, *p* < 0.001) and speech rate (ρ = 0.81, *p* < 0.001), as well as speech rate and VSIL (ρ = 0.845, *p* < 0.001) on the counting task. Weak but significant relationships were observed between *f*_0_ CoV and all timing measures derived from the counting task (ρ > 0.45, *p* < 0.001). Similarly, weak but significant correlations were observed between speech rate derived from the reading task and all other speech metrics (ρ = 0.42–0.46, *p* < 0.001) (with the exception of HNR, which was not significant). HNR co-varied with *f*_0_ CoV on the sustained vowel task (ρ = −0.39, *p* = 0.002) and PSIL on the counting task (ρ = −0.46, *p* < 0.001).

Given the heterogeneity of the data between subjects and speech measures, and the difference in amplitude units, only within-subject correlations were performed to test the relation between amplitude, tremor, and speech measures. The correlations for speech/voice and tremor used both the unilateral and bilateral stimulation conditions and are shown in Figures 1–3. Only data from P2 yielded significant correlations between tremor and speech measures; tremor rating was positively correlated with voice quality and control measures (Figure 3), and two of the four speech timing measures in the counting task (Figure 2). Significant correlations were observed between amplitude and vocal control (*f*_0_ CoV) for P1 (ρ = −0.637, *p* = 0.035) and P4 (ρ = 0.8, *p* = 0.003) and speech timing [P3: SMEAN (reading) ρ = 0.883, *p* = 0.008; P5: speech rate (counting) ρ = 0.783, *p* = 0.013; P6: SMEAN (counting) ρ = −0.648, *p* = 0.031 and PSIL (counting) ρ = −0.653, *p* = 0.029]. Tremor data from P1 significantly correlated with amplitude (ρ = 0.934, *p* < 0.001). Tremor severity did not significantly correlate with amplitude in P2–P6.

### Response to DBS: Patient-specific speech outcomes

P1 showed a monotonic decline in tremor severity with increasing unilateral current. In contrast, P1 showed little variation in speech timing measures on the reading task (Figure 1), with simultaneous deterioration of speech timing at 2 mA on the counting task (Figure 2) and voice quality at 1 mA (Figure 3). For this patient, the optimal stimulus current range (3 mA and above) for tremor suppression did not produce any clear benefits or deficits in speech production.

P2 showed a non-monotonic effect of voltage on tremor suppression, with maximal tremor severity observed at 2 V and minimal severity at 3 V and above. Speech timing measures for the reading passage did not change with voltage (Figure 1); however, all the speech measures derived from the counting and vowel tasks (Figures 2 and 3) followed the same non-monotonic pattern as tremor severity, with the poorest speech outcomes at the same mid-range voltage (2 V). For this patient, DBS stimulation between 2 and 2.5 V caused a deficit in speech production. However, for the stimulus amplitudes that produced the most effective tremor suppression, speech production was similar to the off condition.

P3 also showed a non-monotonic effect of current on tremor suppression, with minimum tremor seen at currents of 1, 2, and 3 mA. Speech timing measures tended to worsen for currents of 4 mA and above (Figure 2). They were similar to the “off” condition for currents that optimized tremor suppression. The vocal control measures (Figure 3) varied non-monotonically with current, with optimum control in the same range as currents producing optimal tremor suppression.

P4 showed only mild tremor suppression due to DBS stimulation, with limited response in speech measures aside from decreased variability of silence length timing at 2 V. The unilateral voltage range for optimal tremor suppression (2 V and above) did not produce any strong speech changes.

P5 showed a trend for more tremor suppression with increasing voltage, with optimal tremor suppression at the highest unilateral voltage. Speech timing measures remained relatively stable on the reading passage (Figure 1); however, changes were observed on the counting task with timing data showing a parabolic pattern with the greatest effect on speech detected between 4 and 5 V. The highest unilateral voltage, at which tremor suppression was optimal, produced speech scores that were equivalent to the “off” condition.

P6 showed little effect of unilateral voltage adjustment, with optimal tremor suppression at the highest voltages (3.6 and 4.3 V) and at 1.5 V. Tremor was completely suppressed, however, in the optimal bilateral condition (downward closed triangle). Speech measures remained relatively stable with changes in unilateral voltage with the exception of speech rate on the counting task, which decreased in line with increased voltage (Figure 2).

In summary, the speech measures did not change consistently with increasing stimulus level, nor were they correlated positively or negatively with the tremor ratings, except for P2. In some cases, the stimulus amplitudes that provided optimal tremor suppression also produced speech scores that were very similar to the “off” condition.

## Discussion

This is a proof of principle study investigating the feasibility of using acoustic measures to quantify speech in response to multiple, randomized amplitude settings in patients receiving DBS for tremor. The preliminary nature of our study was controlled by blinding patients and assessors to stimulation settings; using objective measures of speech; and utilizing a speech battery designed to monitor change by minimizing the impact of practice, familiarity, and fatigue often induced by repetitive production (Vogel et al., 2011; Vogel and Maruff, 2014).

Our data partially support the primary hypothesis that speech outcomes do not correlate with changes in tremor. On a patient by patient basis, only P2 produced speech that statistically significantly co-varied with changes in tremor. The absence of a clear relationship between pitch variability and tremor (except for P2) was surprising given increases in pitch variability (*f*_0_ CoV) can result from poor laryngeal control and vocal tremor. However, inclusion of other markers of vocal tremor, such as intensity variation, may have provided different results (Finnegan et al., 2003). Speech did change as a response to DBS but those changes were not uniform across patients nor were they generally in line with increases in amplitude with the exception of reduced vocal control and increased mean silence length in two patients. PSTA stimulation reduced motor symptoms in the cohort. The lack of a strong relationship between motor and speech function suggest that PSTA stimulation may have dissociable effects on these functions.

In our cohort, optimal experimental tremor suppression was equal to or better than the baseline stimulation settings determined clinically. These data indicate that current/voltage shaping beyond the small stimulation range typically employed in clinical assessments can provide more effective symptom management (Miocinovic et al., 2014). Furthermore, in two cases (P2 and P6), tremor severity at clinical baseline was worse or similar to the “off” condition, suggesting the need for more frequent updating of parameter settings in some individuals (Ruge et al., 2011).

Changes in a narrow range of speech measures corresponding to modifications in amplitude for some patients supports evidence that speech changes with changes in amplitude (Barbe et al., 2014). Three of the four timing measures and 1/2 the voice measures were found to co-vary with changes in amplitude: mean silence length (reading and counting), speech rate (counting), percent silence (counting), and vocal control (vowel). Taken together, these acoustic measures tentatively suggest a subtle but objective change to speech timing and control in 5/6 patients following increases in amplitude. Mean silence length is a measure of pauses between and within words, and increases in this metric can result in dysarthric-like speech. Increases in silence length combined with an overall slowing of speech rate (i.e., fewer syllables produced per second) and increases in variation of fundamental frequency can have an overall detrimental effect on the prosody of speech [prosody in this case defined in terms of suprasegmental components of speech, such as duration, intensity, and *f*_0_ (Fletcher, 2010)]. That is, changes in rate and transitions between the spoken components of speech (as compared to silences between words) can reduce the intelligibility and naturalness of the speaker. When combined with altered voice quality and control, the clarity of a patient’s speech can be wholly undermined by changes in timing; however, vocal control, not voice quality, correlated with changes in amplitude or tremor in some patients.

Recent work exploring the role of amplitude changes and the induction of ataxic-like symptoms has suggested a link between increased stimulation below the thalamus and stimulation-induced ataxia (Herzog et al., 2007; Groppa et al., 2014). Ataxic dysarthria is associated with a slower speech rate, dysphonia (voice quality disturbance), reduced vocal control on sustained vowel tasks, and imprecise articulation (Kent et al., 2000; Folker et al., 2012), among other speech characteristics. Volkman and colleagues (Herzog et al., 2007; Groppa et al., 2014) have proposed the presence of ataxic symptoms may be caused by additional recruitment of adjacent white matter pathways when stimulation amplitudes are increased. Stimulation of the PSTA is known to reduce tremor symptoms (Murata et al., 2003; Hamel et al., 2007; Blomstedt et al., 2010); however, the spread of stimulation beyond the target area may lead to ataxic movement and potentially speech disturbances, thus reducing the overall benefit of DBS. In the current study, strong and statistically significant correlations were differentially found between amplitude and speech timing/vocal control in 5/6 patients. On measures of vocal control (*f*_0_ CoV), P1 had a negative correlation with amplitude, whereas P4 showed the opposite. Similarly, P3 produced greater mean silence lengths on the reading task in line with increases in amplitude, whereas P6 showed the opposite on the counting task. No clear relationship was found between voice quality and amplitude. The heterogeneous response of speech to amplitude and seemingly random relationship with tremor suppression (given that all patients responded to DBS in relation to tremor) highlights the need for more work on determining which clinical/neuroanatomical factors negatively influence speech production following DBS. In spite of the significant correlations between amplitude and speech in the majority of patients, the range of optimal stimulus amplitudes for tremor suppression produced speech measures that were very similar to the device-off condition, suggesting that, at least in these patients, DBS stimulation at *clinically effective* (for tremor suppression) levels did not lead to significant changes in speech overall.

### Implications

Here we present the first study to investigate the effect of randomized amplitude manipulation on speech production by patients using DBS to treat tremor using an objective and repeatable acoustic protocol. It is also one of only a few studies to incorporate objective measures of speech to inform clinical assessment with the aim of devising a simple quantifiable platform on which to base clinical judgments of speech (Mücke et al., 2014). The use of objective acoustic measures allows for quantification of speech changes in conjunction with tremor reduction. We have presented six speech metrics that were automatically acquired using predesigned scripts within a freely available software platform. Pooling variance of these measures suggested that all four speech timing measures were strongly correlated with speech rate in some patients, but not all. For clinicians/researchers seeking an easily interpretable objective measure of speech for DBS optimization, the inclusion of speech rate (syllables per second), voice quality (HNR), and vocal control (*f*_0_ coefficient of variation) derived from the counting and sustained vowel tasks may provide a simplified but objective solution.

The next step in this line of investigation is to develop a speech index that offers the treating clinician a single metric on which to base their decisions on speech production, a *dysarthria index*. The benefit of such an index lies in its potential as a clinical tool with instant quantitative feedback for the treating clinician.

## Limitations

The generalizability of these findings needs to be considered in the context of several methodological caveats. Data were acquired from a small and heterogeneous group of patients. This means that the likelihood of observing stimulation-induced dysarthria was small given that estimates of speech disturbance in essential tremor is around 10% of patients receiving DBS. It appears that only one of the six patients produced speech that changed in line with tremor response to DBS. Altered speech was observed in the remaining five patients to varying degrees. It could be argued that those changes, although present, may not be clinically significant, a hypothesis that needs to be tested in a larger cohort of patients with matched listener ratings. Similarly, a wider array of acoustic measures could be employed to further capture the dysarthria-related changes in production. Candidates could include key spectral and cepstral measures of the voice as well as more targeted metrics derived from hand selected components of speech including slope of second formant transitions as suggested by Weismer et al. (2012). It is important to consider how clinically useful acoustic measures are that require experts to interpret data or time consuming manual analysis. Interpretable data that are acquired and analyzed easily and automatically are necessary for uptake by clinicians.

## Conclusion

The changes in speech observed from DBS amplitude variation are often subtle in isolation. However, the cumulative effect of speech timing, vocal control, and quality deficits can lead to reduced intelligibility and increased dysarthria severity. These side effects can potentially diminish the efficacy of DBS as a tool for improving quality of life. Here we have shown that quantitative analysis of speech can be achieved within the context of evaluating tremor outcomes following randomized amplitude variation. We have also demonstrated that changes in amplitude of stimulation delivered to the PSA can lead to changes in speech but that these changes appear to be patient specific and often not present when optimal settings for tremor suppression were established. Data also suggest that PSTA stimulation may have dissociable effects on speech and other motor functions.

## Conflict of Interest Statement

The authors declare that the research was conducted in the absence of any commercial or financial relationships that could be construed as a potential conflict of interest.
